# Tracking and changes in the clustering of physical activity, sedentary behavior, diet, and sleep across childhood and adolescence: A systematic review

**DOI:** 10.1111/obr.13909

**Published:** 2025-02-18

**Authors:** Finn Blyth, Emma Haycraft, Africa Peral‐Suarez, Natalie Pearson

**Affiliations:** ^1^ School of Sport, Exercise & Health Sciences Loughborough University Loughborough Leicestershire United Kingdom of Great Britain and Northern Ireland; ^2^ Department of Nutrition and Food Sciences Universidad Complutense de Madrid Madrid Spain

**Keywords:** clustering, health behavior, systematic review, young people

## Abstract

**Introduction:**

Clusters of health behaviors (e.g. physical activity/sedentary behavior/diet/sleep) can exert synergistic influences on health outcomes, such as obesity. Understanding how clusters of health behaviors change throughout childhood and adolescence is essential for developing interventions aimed at uncoupling unhealthy behaviors. This review synthesizes prospective studies examining changes in clusters of physical activity, sedentary behavior, diet, and sleep through childhood and adolescence.

**Methods:**

Electronic searches (PubMed, Embase, Web of Science, Scopus) identified prospective studies, published in English up to/including January 2024, of children/adolescents (0‐19 years) which used data‐driven methods to identify clusters of 2/more behaviors (physical activity, sedentary behaviors, diet, sleep) at multiple timepoints. A narrative synthesis was conducted due to methodological heterogeneity.

**Results:**

Eighteen studies reporting data from 26,772 individual participants were included. Eleven studies determined clusters at each timepoint (i.e. identified clusters at T1 and T2, respectively), while seven determined clusters longitudinally using behavioral data across multiple timepoints. Among studies that identified clusters at each timepoint, participants commonly transitioned to similarly characterized clusters between timepoints. Where cluster tracking was examined, 64% of clusters had stable transition probabilities of 60‐100%. The most prevalent longitudinal cluster trajectories were characterized by co‐occurring healthy behaviors which remained stable. Remaining within unhealthy clusters at multiple timepoints was associated with higher markers of adiposity.

**Conclusion:**

‘Healthy’ and ‘unhealthy’ clusters remained highly stable over time, suggesting behavioral patterns developed early can become entrenched and resistant to change. Interventions focused on instilling healthy behaviors early are required to provide a strong foundation for behavioral stability throughout life.

## 1 INTRODUCTION

The prevalence of children and adolescents living with obesity is concerning across a wide range of countries.^1^ There is a wealth of evidence suggesting that modifiable health behaviors such as physical activity, sedentary behavior, diet, and sleep are key contributors to an increased risk of obesity among children and adolescents.^2^, ^3^, ^4^, ^5^ For example, recent research found that 51% of boys and 43% of girls aged 8‐10 had three or more behavioral risk factors associated with obesity.^6^ Insufficient physical activity and excessive sedentary behavior are widespread among children and adolescents around the world.^7^, ^8^ There are also concurrent global indications of unfavorable changes in dietary and sleep patterns as children age such as declines in overall sleep duration and increased fast food consumption.^9^, ^10^ Research has shown that individual health behaviors develop in childhood and track through adolescence and into adulthood,^11^, ^12^, ^13^, ^14^, ^15^ and thus promoting optimum health behaviors in childhood is important for current and future health.

Much research on health behaviors has tended to focus on these behaviors in isolation, however, a recent body of research has shown that health behaviors such as physical activity, sedentary behavior, diet, and sleep tend to co‐occur or ‘cluster’ within groups of individuals.^16^, ^17^, ^18^, ^19^ Research indicates that the clustering of unhealthy behaviors exerts synergistic influences on both physical (e.g. obesity) and mental (e.g. depression) health outcomes.^20^, ^21^, ^22^, ^23^ In other words, the cumulative impact of multiple unhealthy behaviors is more detrimental to health than the effects of each behavior individually.^24^

Understanding how, and in who, such behaviors cluster is an important step in identifying targets for public health interventions. Targeted interventions that successfully induce changes in multiple behaviors have not only shown to be more cost‐effective, but they also have an amplified impact, benefiting those who are most in need.^25^, ^26^, ^27^

The clustering of domains of physical activity, sedentary behavior, diet, and sleep, however, is complex and currently not very well understood.^28^ Much of the evidence to date comes from cross‐sectional studies^29^, ^30^, ^31^, ^32^, ^33^, ^34^ which have found that combinations of these behaviors cluster in both healthy and unhealthy ways and that the profiles of clusters vary across different socio‐demographic groups.^21^, ^28^, ^35^, ^36^ A recent review^35^ identified differences between the types of clustering present in younger people (for example, children and young adolescents) compared to older adolescents and young adults, and among those from higher socioeconomic status compared to those from lower socioeconomic status backgrounds. Unhealthy or mixed clusters characterized by low physical activity were more prevalent among older children and those from lower socioeconomic backgrounds, whereas healthy or mixed clusters characterized by higher physical activity were more prevalent among younger children and those from higher socioeconomic background.^35^ These findings are consistent with previous reviews examining sociodemographic characteristics of clusters of health behaviors.^21^, ^22^, ^28^, ^36^

While the synthesis of cross‐sectional evidence is useful for understanding cluster profiles and key sociodemographic determinates of clusters of health behaviors, there is a need to better understand how clusters of behaviors evolve over time. Such evidence is needed to improve our understanding of the trends in health behavior clustering and to highlight critical time points where behaviors may start to change and cluster differently. Understanding how different cluster patterns track or change across the key developmental periods of childhood and adolescence is particularly important. This information would be invaluable for identifying which behaviors need to be simultaneously targeted and which population subgroups are most at risk for the development and persistence of less healthy behaviors.

Previous systematic reviews have identified several key gaps in our understanding of health behavior clustering in children and adolescents. For instance, while there is a common focus on studying the clustering of physical activity and sedentary behavior in young people, there is comparatively less exploration of the clusters of these behaviors alongside aspects of the diet or sleep.^28^, ^35^, ^36^ Research has highlighted the synergistic effects of combinations of unhealthy levels of physical activity, sedentary behavior, diet, and sleep in causing premature mortality^37^, ^38^, ^39^ stressing the need to direct efforts at exploring clustering of these specific behaviors.

Therefore, the aim of this systematic review is to synthesize the evidence of prospective studies examining the transitions and/or trajectories of clusters of physical activity, sedentary behavior, diet, and sleep through childhood and adolescence. This review also aimed to synthesize the evidence regarding a) the sociodemographic determinants of cluster transitions and b) the associations between clusters and mental and physical health outcomes.

## 2 MATERIALS & METHODS

This review was registered with the International Prospective Register of Systematic Reviews (PROSPERO) (CRD42023382681) and is reported in accordance with the Preferred Reporting Items for Systematic Reviews and Meta‐Analyses (PRISMA).^40^

### 2.1 Search strategy

A comprehensive search strategy was developed using key terms for Population (children and adolescents aged 0‐19), health behaviors ‐ i.e. physical activity, sedentary behavior, diet, and sleep, and change in clusters over time (e.g. change, trajectories).

Preliminary searches were used to refine the search strategy and results were checked against a list of studies identified in a preliminary scoping review to ensure reliability. The finalized search strategy can be found in supplementary file1. Relevant studies were identified though searches of the electronic databases PubMed/MEDLINE, Web of Science, Scopus, and Embase conducted up to and including January 2024. In addition, we cross‐checked the results of the electronic searches with reference lists of recent systematic reviews of health behavior clustering.^28^, ^35^, ^36^

### 2.2 Inclusion and exclusion criteria

To be eligible for this review studies had to: (1) have used data‐driven methods to identify clusters (e.g. cluster analysis or Latent Class Analysis) of two or more of the following health behaviors: physical activity, sedentary behaviors, diet, or sleep; (2) be longitudinal/prospective in design (e.g. cohort studies or the control arm of a randomized control trial), and report information on the health behaviors of interest in the same participants on at least two separate time points; (3) include children and adolescents aged 0‐19 at baseline as participants of the study; (4) be published in English up to and including January 2024.

### 2.3 Identification of relevant studies

This systematic review was managed using Covidence, a systematic review management software. All studies retrieved during the electronic searches were imported into Covidence where duplicate records were automatically removed. Two independent reviewers (out of FB, APS, NP) initially screened titles and abstracts for eligibility. Following this, two reviewers independently screened each of the full‐text articles for eligibility (FB, APS). Conflicts between reviewers were resolved by a third member of the review team (NP or EH).

### 2.4 Data extraction

A standardized data extraction form was developed in Microsoft Excel. This form was piloted by the review team for suitability and refined. The following information was extracted from each study: (1) general information ‐ author, year, study title, country; (2) study characteristics ‐ sample size, number of independent samples, characteristics of independent samples, socio‐demographic variables, socio‐demographic variables assessment method, number of assessment points for clusters, baseline age, time between assessments; (3) behavioral data ‐ physical activity measure and exposure, sedentary behavior measure and exposure, dietary behavior measure and exposure, sleep measure and exposure; (4) outcomes ‐ health outcomes assessed, health outcome assessment method, type of cluster patterning examined, cluster assessment method, clusters identified, method of analysis (associations between sociodemographic variables and trajectories), method of analysis (associations between trajectories and health outcomes); (5) main findings.

### 2.5 Risk of bias assessment

A risk of bias assessment was completed for all included studies, using the Cochrane risk of bias assessment tool for observational studies.^22^ Each included study was assessed against the following domains: 1) selection bias, 2) performance bias, 3) detection bias, 4) attrition bias, 5) selective reporting bias, and 6) other factors that may increase the risk of bias. Within each of these domains, studies were classified as either a low risk of bias, a high risk of bias, or an unclear risk of bias. Each study was assessed independently by two separate reviewers (FB, APS) and conflicts were discussed and resolved with a third member of the review team (NP).

### 2.6 Data synthesis

For synthesis, studies were tabulated based on how they examined clusters of behaviors over time. Two types of studies were identified: 1) studies that identified clusters cross‐sectionally at multiple timepoints. Studies in this category created one set of clusters using baseline data and then another set of clusters using data at follow‐up(s), and examined how participants tracked (i.e. remaining in the same cluster type at baseline and at follow‐up) or changed (i.e. changed from one cluster at baseline to a different cluster type at follow up.) over time. For the purpose of this review, these were labeled ‘Transition’ studies; 2) studies where data from multiple time‐points was simultaneously analyzed to identify longitudinal clusters among individuals who share similar trajectories of health behaviors across time. For the purpose of this review, these studies were labeled ‘Trajectory’ studies.

For studies examining ‘Transitions’, the primary measure used to convey the likelihood of tracking or change between clusters was transition probabilities. Transition probabilities help model the dynamics of systems that change over time, providing insights into the prospects of transitions between different states. Where possible these transition probabilities have been converted into percentages and reported in tables. In some studies, the same types of clusters were observed at baseline and follow‐up allowing for tracking to be examined, however, other studies found different types of clusters at different timepoints, which precluded their capacity to examine tracking. Thus, in these cases, only change has been reported. The clusters generated in studies of ‘Trajectories’ are longitudinal in nature and, as such, the way in which change is conveyed is by reporting the prevalence of these clusters among samples.

Labels of clusters and summaries have been listed in tables as they are reported in the original articles. In cases where analysis of clusters was performed according to subgroups (e.g. boys and girls), the results have been presented according to independent samples.

Due to significant heterogeneity in the type of cluster patterning examined, the methods and measures of individual behaviors entered into cluster analyses, and the methods used to determine clusters across the studies, a meta‐analysis was deemed not to be viable. Results have therefore been synthesized narratively.

## 3 RESULTS

**FIGURE 1:**
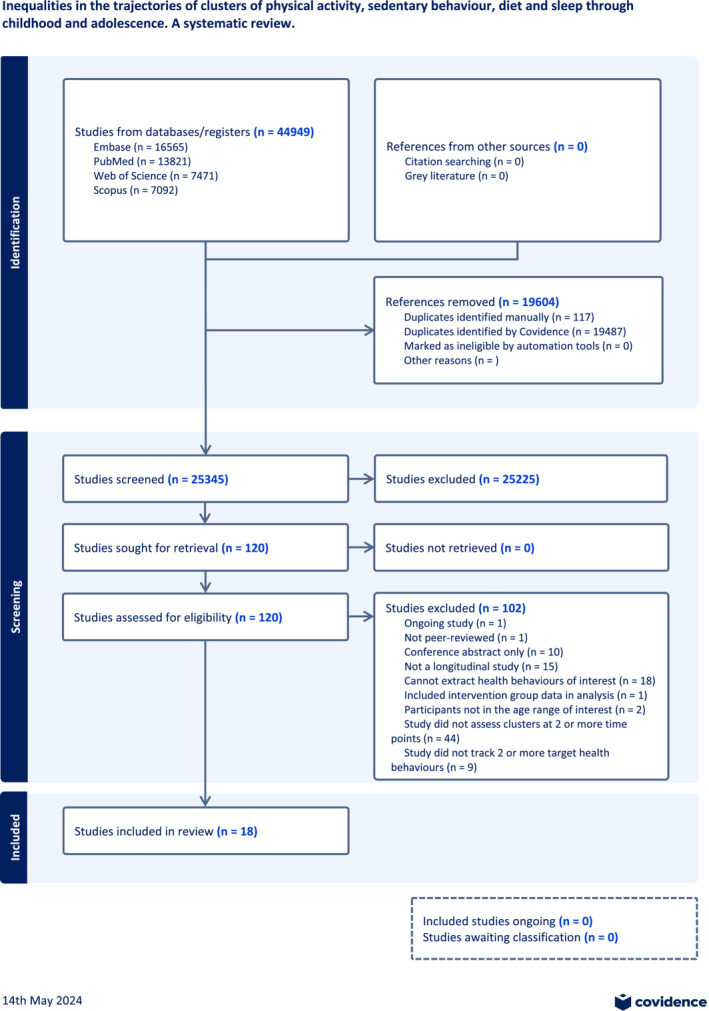
PRISMA flowchart titled ‘Inequalities in the trajectories of clusters of physical activity, sedentary behavior, diet and sleep through childhood and adolescence. A systematic review.’ This flow chart created with Covidence outlines the screening process.

Searches of electronic databases yielded 44,949 records of which 18 studies^41^, ^42^, ^43^, ^44^, ^45^, ^46^, ^47^, ^48^, ^49^, ^50^, ^51^, ^52^, ^53^, ^54^, ^55^, ^56^, ^57^, ^58^ were eligible for this review. A complete summary of the search process is presented in the PRISMA flowchart (Figure 1).

### 3.1 Study characteristics

Table 1 displays the characteristics of the 18 included studies. Studies were identified from across six continents, with most studies coming from Oceania (n = 7),^42^, ^47^, ^50^, ^54^, ^55^, ^56^, ^57^ and Europe (n = 6).^41^, ^43^, ^44^, ^46^, ^49^, ^51^ Two studies were from North America,^48^, ^52^ two from Asia,^45^, ^58^ and one from Africa.^53^ Studies were published between 2015 and 2024, with most (n = 16, 89%) published in the last five years. Sample sizes ranged from 87 to 4164. Baseline ages ranged from 0‐1 years^55^ to 16.9 years^50^ and half of the studies (n = 9) were conducted with children aged between 5 and 11 years at baseline. Nine studies^44^, ^45^, ^46^, ^49^, ^50^, ^53^, ^55^, ^57^, ^58^ examined sociodemographic determinates of clusters, and six^41^, ^42^, ^43^, ^49^, ^55^, ^58^ examined the association between clusters and health outcomes.

**TABLE 1:** Characteristics of studies included in the review.

Sample size:	References:
<500	^42^ ^(Y),^ ^42^ ^(O)^^,^ ^49^, ^52^ ^(B)^^,^ ^54^ ^(i)^^,^ ^54^ ^(ii),^ ^56^
500 – 1000	^41^ ^(B)^^,^ ^41^ ^(G),^ ^44^, ^47^, ^50^, ^52^ ^(G)^^,^ ^53^ ^(B)^^,^ ^53^ ^(G)^ ^58^
1000 – 1500	^43^, ^45^, ^48^ ^(B)^^,^ ^48^ ^(G)^^,^ ^57^ ^(B)^^,^ ^57^ ^(G)^
>1500	^46^, ^51^, ^55^ ^(Y)^^,^ ^55^ ^(O)^
**Number of follow‐ups where clustering has been assessed:**	
1	^41^ ^,^ ^50^, ^54^ ^(i),^ ^56^
2	^49^ ^,^ ^51^ ^,^ ^53^ ^,^ ^54^ ^(ii)^^,^ ^55^, ^57^, ^58^
**Duration of follow ups:**	
≤1 Year	^48^, ^51^, ^53^
≤ 2 Years	^43^, ^45^, ^46^, ^49^, ^50^, ^55^, ^57^
≤ 3 Years	^41^, ^42^, ^44^, ^47^, ^54^, ^56^, ^58^
**Baseline age:**	
<5 years	^41^, ^54^, ^55^, ^56^, ^58^
5‐11 years	^42^ ^(Y),^ ^44^, ^45^, ^47^, ^48^, ^49^, ^51^, ^52^, ^57^
12‐18 years	^42^ ^(O),^ ^43^, ^46^, ^50^, ^53^
**Gender**	
Boys and girls combined	^42^ ^,^ ^43^ ^,^ ^44^, ^45^ ^,^ ^46^, ^47^, ^49^ ^,^ ^50^ ^,^ ^51^, ^54^, ^55^, ^56^, ^58^
Boys and girls separately	^41^ ^,^ ^48^, ^52^, ^53^, ^57^
**Outcomes included in studies**	
Physical activity	^41^, ^42^, ^43^, ^44^, ^45^, ^46^, ^47^, ^48^, ^49^, ^50^, ^51^, ^52^, ^53^, ^54^, ^55^, ^56^, ^57^, ^58^
Sedentary behavior	^41^, ^42^, ^43^, ^44^, ^45^, ^46^, ^47^, ^48^, ^49^, ^50^, ^51^, ^52^, ^53^, ^54^, ^55^, ^56^, ^57^, ^58^
Diet	^41^, ^42^, ^46^, ^47^, ^54^, ^56^, ^58^
Sleep	^41^, ^45^, ^47^, ^52^, ^53^, ^56^, ^57^, ^58^
**Physical activity outcome**	
MVPA	^42^, ^43^, ^44^, ^45^, ^46^, ^49^, ^50^, ^51^, ^52^, ^56^, ^57^, ^58^
Light physical activity	^44^, ^45^, ^57^
Vigorous physical activity	^45^, ^46^, ^50^
Inactivity	^45^
Outdoor play time	^41^, ^54^, ^56^, ^58^
Organized sport participation	^41^, ^47^, ^48^, ^53^, ^56^, ^58^
Walking time	^41^, ^46^, ^50^
Frequent physical exertion	^48^
Daily free play	^48^
Active travel	^41^, ^50^
Physical activity	^53^, ^55^
Physical activities done with mother/father	^41^
Physical education	^53^
**Assessment of Physical activity outcome**	
Accelerometer	^42^, ^43^, ^44^, ^45^, ^49^, ^51^, ^56^
Time use diaries	^55^, ^57^
Self‐report questionnaires	^46^, ^48^, ^52^, ^53^
Parent‐report questionnaires	^41^, ^47^, ^54^, ^56^, ^58^
**Sedentary behavior outcome**	
Sedentary time	^42^, ^43^, ^44^, ^46^, ^47^, ^48^, ^49^, ^51^, ^53^, ^56^, ^57^
Screen time	^47^, ^52^, ^55^, ^56^, ^58^
Television viewing	^41^, ^42^, ^50^, ^54^
Television viewing during meals	^41^
Video gaming	^47^, ^50^, ^56^
Computer use	^50^
Quiet Play	^47^, ^56^
**Assessment of sedentary behavior outcome**	
Accelerometer	^42^, ^43^, ^44^, ^47^, ^49^, ^51^, ^56^
Time use diaries	^55^, ^57^
Self‐report questionnaires	^46^, ^47^, ^48^, ^50^, ^52^, ^53^
Parent‐report questionnaires	^41^, ^42^, ^54^, ^56^, ^58^
**Dietary behavior outcome**	
Fruit and Vegetable intake	^42^, ^46^, ^47^, ^54^, ^56^, ^58^
Dairy product intake	^41^, ^56^
Sugary drink consumption	^41^, ^54^, ^58^
Sugar product intake	^47^, ^54^, ^56^, ^58^
Savory food intake	^47^, ^56^, ^58^
Starchy food intake	^46^
Salty and fatty food intake	^46^
Energy dense food/drink consumption	^42^
Water intake	^54^
Baby food intake	^41^
Snacking	^41^, ^46^
Breakfast consumption	^41^
Frequency of discretionary food items consumed	^41^, ^47^, ^54^
Processed meat consumption	^58^
Fast food consumption	^58^
**Measure of dietary outcome**	
Self‐report food frequency questionnaires	^46^
Parent‐report food frequency questionnaires	^41^, ^42^, ^47^, ^54^, ^56^, ^58^
**Sleep outcome**	
Night sleep duration	^41^, ^45^, ^47^, ^52^, ^56^, ^57^, ^58^
School night sleep	^53^
Weekend sleep	^53^
Napping	^57^
**Assessment of sleep outcome**	
Accelerometer	^45^
Time use diaries	^57^
Self‐report questionnaires	^52^, ^53^
Parent‐report questionnaires	^41^, ^47^, ^56^, ^58^
**Method used to Identify clusters**	
Cluster analysis	^42^
Principle component analysis	^54^
Latent class analysis	^41^, ^44^, ^47^, ^53^
Latent transition (profile) analysis	^43^, ^45^, ^46^, ^48^, ^50^
Parallel process growth mixture modeling	^51^
Multi‐group trajectory modeling	^49^, ^52^, ^55^, ^57^, ^58^
**Type of cluster patterning examined:**	
Transitions	^41^, ^42^, ^43^, ^44^, ^45^, ^46^, ^47^, ^48^, ^50^, ^54^, ^56^
Trajectories	^49^, ^51^, ^52^, ^53^, ^55^, ^57^, ^58^
**Sociodemographic determinates assessed in association with change/tracking/trajectories of clusters:**	
Gender	^44^, ^45^, ^46^, ^49^, ^55^, ^56^, ^58^
Ethnicity	^45^, ^58^
Indigenous status	^55^
Indicators of socio‐economic status	^41^, ^44^, ^46^, ^53^, ^54^, ^55^, ^56^, ^57^, ^58^
Post‐secondary education situational pathways.	^50^
Age	^46^, ^56^
Child's birth order	^53^, ^58^
Number of siblings	^55^
English spoken at home	^55^
Mother country of birth	^53^
Living with two biological parents	^55^
**Health outcomes of change/tracking/trajectories of clusters assessed:**	
Indicators of adiposity	^41^, ^49^, ^57^, ^58^
Weight status	^49^, ^57^
HRQOL	^43^, ^55^
Positive affect	^43^
Negative affect	^43^
Socio‐emotional outcomes	^55^
Blood pressure	^58^
HDL cholesterol	^58^
Fasting insulin	^58^
Triglyceride	^58^
HOMA‐IR	^58^
Fasting glucose	^58^
Fasting insulin	^58^
Metabolic syndrome score	^58^
Fatty liver index	^58^
**Region:**	
Europe	^41^, ^43^, ^44^, ^46^, ^49^, ^51^
North America	^48^, ^52^
South America	
Africa	^53^
Oceania	^42^, ^47^, ^50^, ^54^, ^55^, ^56^, ^57^
Asia	^45^, ^58^

G, girls only, B, boys only, BG, boys and girls combined, Y, younger children, O, older children, T, timepoint, HRQOL, health‐related quality of life.

References: Saldanha‐Gomes et al (2020)^41^; Leech et al (2015^42^); Sánchez‐Oliva et al (2020)^43^; Jago et a., (2018)^44^; Padmapriya et al (2021^45^); Dakin et al (2021^46^); D'Souza et al (2021)^47^; Clark et al (2022)^48^; Farooq et al (2021^49^); Parker et al. (2022)^50^; Parker et al (2021)^51^; Gallant et al. (2020)^52^; Hanson et al (2019)^53^; Lioret et al (2020^54^); Del Pozo‐Cruz et al (2019^55^); D'Souza et al (2023)^56^; Wilhite et al. (2023)^57^; Chia et al (2024)^58^

### 3.2 Risk of bias and quality assessment

**FIGURE 2:**
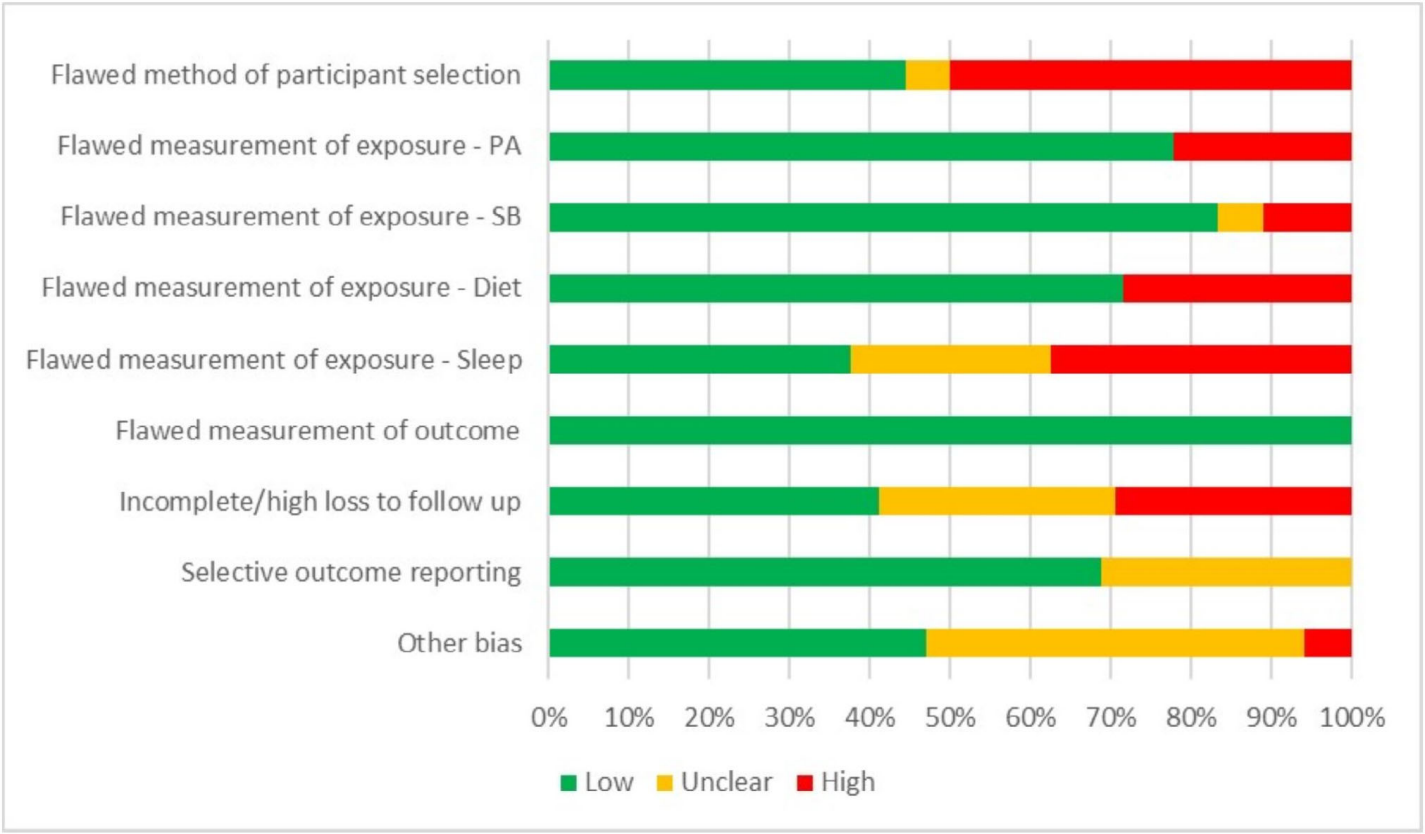
Stacked bar chart titled ‘Overview of study quality and risk of bias [low, high, and unclear] assessment (n=18 studies)’. This chart provides an overview of study quality and risk of bias.

Most (n = 15, 83%) studies had at least one high‐risk judgment score across the risk of bias domains^41^, ^42^, ^43^, ^44^, ^45^, ^46^, ^47^, ^50^, ^51^, ^52^, ^53^, ^54^, ^55^, ^56^, ^58^ (Figure 2). Two‐thirds (67%) of studies had at least one unclear judgment due to lack of information.^41^, ^42^, ^43^, ^44^, ^45^, ^47^, ^48^, ^53^, ^55^, ^56^, ^57^, ^58^ All studies had at least one low‐risk judgment. Fifteen studies (83%) had an overall high‐risk judgment,^41^, ^42^, ^43^, ^44^, ^45^, ^46^, ^47^, ^50^, ^51^, ^52^, ^53^, ^54^, ^55^, ^56^, ^58^ two (11%) studies had an overall unclear judgment^48^, ^57^ and one study (6%) had an overall low‐risk judgment.^49^

### 3.3 Overview of behaviors assessed

Six studies (33%) used accelerometers to measure physical activity,^42^, ^43^, ^44^, ^45^, ^49^, ^51^ four (22%) used participant self‐report questionnaires,^46^, ^48^, ^52^, ^53^ four (22%) used parent‐report questionnaires,^41^, ^47^, ^54^, ^58^ and one study (6%) used time use diaries to measure physical activity.^55^ Sedentary behavior was measured using accelerometers in six studies,^42^, ^43^, ^44^, ^47^, ^49^, ^51^ self‐report questionnaires in six studies,^46^, ^47^, ^48^, ^50^, ^52^, ^53^ parent‐report questionnaires in four studies,^41^, ^42^, ^54^, ^58^ and time use diaries in one study.^55^ Diet was measured using parent‐report food frequency questionnaires in five studies,^41^, ^42^, ^47^, ^54^, ^58^ and participant self‐report food frequency questionnaires in one study.^46^ Sleep was measured using participant self‐report questionnaires in two studies,^52^, ^53^ parent‐report questionnaires in three studies,^41^, ^47^, ^58^ and accelerometers in one study^45^ (Table 1).

Physical activity and sedentary behavior were the most common behaviors examined and were included in all 18 papers. Seven studies (39%) examined sleep,^41^, ^45^, ^47^, ^52^, ^53^, ^56^, ^57^ and six studies (33%) examined diet.^41^, ^42^, ^46^, ^47^, ^54^, ^56^ Across the 18 included studies, 13 different physical activity behavioral outcomes were identified (e.g. organized sports participation), seven sedentary behavioral outcomes (e.g., screen time), 15 dietary behavioral outcomes (e.g. fruit and vegetable intake), and four sleep outcomes (e.g. night sleep duration).

### 3.4 Cluster analysis methods

Eleven studies (61%) examined transitions between clusters over time^41^, ^42^, ^43^, ^44^, ^45^, ^46^, ^47^, ^48^, ^50^, ^54^, ^56^ (see Table 2). Nine of these studies (50%) used latent class analysis (LCA) and/or latent transition analysis (LTA) to determine clusters. One study (6%)^54^ used principal component analysis (PCA), and one study (6%)^42^ used a K‐means cluster analysis to determine clusters.

**TABLE 2:** Transitions between health behavior clusters across time, and associations with health outcomes. This table provides an in‐depth overview of studies in the ‘tracking’ category.

Author (year)	Clusters identified (%) and summary (baseline)	Clusters identified (%) and summary (follow up)	Changes in cluster prevalence across time	Outcomes assessed	Results (outcomes)
**<5 years**					
D'Souza et al, (2023)	C1: Unhealthy (21.4%), *MVPA ↓ SG↑ FV↓ SP↓* C2: Non‐sedentary healthy eaters (57.9%), *ST↓ FV↑ SG↓* C3 Active unhealthy eaters (20.8%), *MVPA↑ OP↑ SB↓ SG↑ SV↑ FV↓*	C1: Unhealthy (18.6%), *MVPA↓ SB↑ FV↓ SP↓* C2: Intermediate (79.3%); *OP‐ OS‐ MVPA‐ SB‐ ST‐ FV‐ SG‐ SV‐ SP‐* C3: Active and non‐sedentary (2.1%); *OP↑ OS↑ MVPA↑ SB↓*	C1 → C1 (43.1%) C1 → C2 (55.5%) C1 → C3 (1.4%) C2 → C1 (9.7%) C2 → C2 (75.4%) C2 → C3 (14.9%) C3 → C1 (2.9%) C3 → C2 (50.0%) C3 → C3 (47.1%)	BMI z‐score Waist circumference	The highest BMI z‐score and the largest waist circumference were observed for those in the unhealthy pattern at both timepoints.
Lioret et al. (2020)	**Independent sample 1:** C1: “Discretionary consumption and TV”, *SG↑ SV↑ TV↑* C2: “Fruit, vegetables and outdoor” *FV↑ OPA↑*	**Independent sample 1 (T2):** C1: “Discretionary consumption and TV”, *SG↑ SV↑ TV↑* C2: “Fruit, vegetables and outdoor” *FV↑ OPA↑*	**T1 ‐ T2** **C1 → C1 (stable) (r = 0.52)** C1 → C2 (r = −0.20) C2 → C1 (r = −0.05) **C2 → C2 (stable) (r = 0.47)** **T1 ‐ T3** **C1 → C1 (stable) (r = 0.50)** C1 → C2 (r = −0.17) C2 → C1 (r = −0.12) **C2 → C2 (stable) (r = 0.36)** **T2 ‐ T3** **C1 → C1 (stable) (r = 0.63)** C1 → C2 (r = −0.13) C2 → C1 (r = −0.23) **C2 → C2 (stable) (r = 0.60)**		
		**Independent sample 1 (T3):** C1: “Discretionary consumption and TV”, *SG↑ SV↑ TV↑* C2: “Fruit, vegetables and outdoor” *FV↑ OPA↑*			
	**Independent sample 2:** C1: “Discretionary consumption and TV”, *SG↑ SV↑ TV↑* C2: “Fruit, vegetables and outdoor” *FV↑ OPA↑*	**Independent sample 2: (T2)** C1: “Discretionary consumption and TV”, *SG↑ SV↑ TV↑* C2: “Fruit, vegetables and outdoor” *FV↑ OPA↑*	**T1 – T2** **C1 → C1 (r = 0.51)** C1 → C2 (r = −0.06) C2 → C1 (r = −0.04) **C2 → C2 (r = 0.51)**		
Saldanha‐Gomes et al (2020)*	**Boys:** C1: “Unhealthy eating cluster (40.1%)”, *DT↓* C2: “Healthy eating cluster (59.9%), *DT ↑*	**Boys:** C1: “High TV‐unhealthy eating cluster (53.3%)” *TV↑ DT↓* C2: “Low TV‐healthy eating cluster (46.7%)”, *TV↓ DT↑*	C1 → C1 (n = 148) C1 → C2 (n = 64) C2 → C1 (n = 122 C2 → C2 (n = 177)	Body fat percentage	0
	**Girls:** C1: “Unhealthy eating cluster (45.9%)”, *DT↓* C2: “Healthy eating cluster (54.1%)” *DT ↑*	**Girls:** C1 “Very low TV‐low outdoor PA cluster (30.4%)”“; *TV↓* ^*+*^ *PA↓* C2: “Moderate TV‐high outdoor PA cluster (19.8%)”, *TV‐ PA↑* C3: “High TV‐low outdoor PA cluster (43.1%)”, *TV↑ PA↓* C4: “Very high TV‐high outdoor PA’ (6.8%)” *TV↑* ^*+*^ *PA↑*	C1 → C1 (n = 20) C1 → C2 (n = 92) C1 → C3 (n = 37) C1 → C4 (n = 33) C2 → C1 (n = 8) C2 → C2 (n = 98) C2 → C3 (n = 50) C2 → C4 (n = 102)	Body fat percentage	Girls belonging to a cluster evolution path characterized by continued high TV exposure and an unfavorable mealtime setting from age 2 had a higher body fat percentage at age 5
**5‐11 years**					
Clark et al. (2022)	**Males:** C1: “Active Screeners “, *PA↑ SB↑* C2: “Low sedentary behaviors”, *PA↑ SB↓ ST↓* C3: “Sedentary/inactive” *PA↓ SB↑*	**Males:** C1: “Active Screeners “, *PA↑ SB↑* C2: “Low sedentary behaviors”, *PA↑ SB↓ ST↓* C3: “Sedentary/inactive” *PA↓ SB↑*	**C1 → C1 (stable) (87.9%)** C1 → C2 (0.0%) C1 → C3 (12.1%) C2 → C1 (1.3%) **C2 → C2 (stable) (89.9%)** C2 → C3(8.8%) C3 → C1 (6.9%) C3 → C2 (0.8%) **C3 → C3 (stable) (92.3%)**		
	**Females:** C1: “Active Screeners”, *PA↑ SB↑* C2: “Low sedentary behaviors “, *PA↑ SB↓ ST↓* C3: “Sedentary/inactive” *PA↓ SB↑*	**Females:** C1: “Active Screeners”, *PA↑ SB↑* C2: “Low sedentary behaviors “, *PA↑ SB↓ ST↓* C3: “Sedentary/inactive” *PA↓ SB↑*	**C1 → C1 (stable) (88.1%)** C1 → C2 (0.0%) C1 → C3 (11.9%) C2 → C1 (10.4%) **C2 → C2 (stable) (88.5%)** C2 → C3(1.2%) C3 → C1 (7.9%) C3 → C2 (1.2%) **C3 → C3 (stable) (90.9%)**		
D'Souza et al. (2021) **	C1: “Unhealthy (20.6%)”, *PA↓ SB↑ FV↓ SG↑ SP ↓* C2: “Non‐sedentary healthy eaters (57.3%)”, *ST ↓ FV↑ SG↓* C3: “Active unhealthy eaters (22.1%)”, *PA↑ ST↓ FV↓*	C1: “Unhealthy (18.6%)”, *PA↓ ST↑ VG↑ SB↑ FV↓ SP↓* C2: “Intermediate (64.3%)” *PA‐ ST‐ VG‐ SB‐ FV‐ SG‐ SP‐* C3: “Active and non‐sedentary (17.1%)” *PA↑ ST↓*	Change not assessed		
Jago et al, (2018)	C1: “Highly Active (9%)”, *PA↑* ^*+*^ *SB↓* C2: “Active/Light (29%)”, *PA↑ SB‐* C3: “Active/Sed (19%)”, *PA↑ SB↑* C4: “Inactive/Light (15%)”, *PA↓ SB‐* C5: “Inactive/Sed (28%)” *PA↓ SB↑*	C1: “Highly active (7%)”, *PA↑* ^*+*^ *SB↓* C2: “Active/light (6%)”, *PA↑ SB‐* C3: “Active/sed (11%)”, *PA↑ SB↑* C4: “Average (33%)”, *PA‐ SB‐* C5: “Inactive/Light (22%):”, *PA↓* ^*+*^ *SB‐* C6: “Inactive/Sed (21%)” *PA↓ SB↑* ^*+*^	C1 → C1 (39%) C1 → C2 (21%) C1 → C3(4%) C1 → C4 (16%) C1 → C5 (15%) C1 → C6 (5%) C2 → C1 (8%) C2 → C2 (5%) C2 → C3 (2%) C2 → C4 (70%) C2 → C5 (12%) C2 → C6 (3%) C3 → C1 (7%) C3 → C2(1%) C3 → C3 (43%) C3 → C4 (19%) C3 → C5 (0%) C3 → C6 (30%) C4 → C1 (2%) C4 → C2 (5%) C4 → C3(3%) C4 → C4 (21%) C4 → C5 (67%) C4 → C6 (2%) C5 → C1 (0%) C5 → C2 (5%) C5 → C3 (8%) C5 → C4 (14%) C5 → C5 (25%) C5 → C6 (48%)		
Leech et al (2015) (Younger children) ***	C1: “Mostly healthy (40%)”, *PA↑ EDC↓ ST↓ TV↓* C2: “ED consumers who watch TV (35%)”, *EDC↑ TV ↑* C3: “High sedentary behavior/low MVPA (25%) *PA↓ ST↑*	C1: “Mostly healthy (34%)”, *PA↑ EDC↓ ST↓ TV↓* C2: “ED consumers who watch TV (25%)”, *EDC↑ TV ↑* C3: “High sedentary behavior/low MVPA (41%) *PA↓ ST↑*	**C1 → C1 (stable) (49%)** C1 → C2 (3%) * C1 → C3 (17%) * C2 → C1 (11%) * **C2 → C2 (stable) (42%)** C2 → C3(9%)* C3 → C1 (3%* C3 → C2 (7%) * **C3 → C3 (stable) (58%)**		
Padmapriya et al (2021)	C1: “Rabbits (12.4%)” *PA↑↑ SB↓ SP‐* C2: “Chimpanzees (51.1%)” *PA↑ SB↓ SP‐* C3: “Pandas (24.4%)” *PA↓ SB↑ SP↑* C4: “Owls (12%)” *PA↓ SB↑ SP↓*	C1: “Rabbits (16.5%)”, *PA↑↑ SB↓ SP‐* C2: “Chimpanzees (39.1%)” *PA↑ SB↓ SP‐* C3: “Pandas (36.7%)”, *PA↓ SB↑ SP*↑ C4: “Owls (7.7%)” *PA↓ SB↑ SP↓*	**C1 → C1 (stable) (81%)** C1 → C2 (12%) C1 → C3 (5%) C1 → C4 (2%) C2 → C1 (10%) **C2 → C2 (stable)(61%)** C2 → C3 (27%) C2 → C4 (2%) C3 → C1 (2%) C3 → C2 (20%) **C3 → C3 (stable) (59%)** C3 → C4 (19%) C4 → C1 (6%) C4 → C2 (14%) C4 → C3 (56%) **C4 → C4 (stable) (24%)**		
**12‐18 years**					
Dakin et al. (2021)	C1: “Healthy diet and high PA (7.9%)”, *FV↑ SG↓ FS↓ NB↓ PA↑* C2: “Big eater and moderate to high PA (23.8%)”, *FV↑ ST↑ DA↑ SG↑ FS↑ NB↑ PA↑* C3: “Healthy diet and low PA (31.2%)”, *SG↓ FS↓ NB↓ PA↓* C4: “Restrictive diet and moderate PA (20.6%)”, *FV↓ ST↓ DA↓ FS↓ PA‐* C5: “Sugar products, nibbling and moderate PA (16.5%)” *SG↑ NB↑ PA‐*	C1: “Healthy diet and high PA (10.8%)”, *FV↑ SG↓ FS↓ NB↓ PA↑* C2: “Big eater and moderate to high PA (23.7%)”, *FV↑ ST↑ DA↑ SG↑ FS↑ NB↑ PA↑* C3: ‘Healthy diet and low PA (31.3%)”, *SG↓ FS↓ NB↓ PA↓* C4: “Restrictive diet and moderate PA (19.2%)”, *FV↓ ST↓ DA↓ FS↓ PA‐* C5: “Sugar products, nibbling and moderate PA (14.9%)” *SG↑ NB↑ PA‐*	**C1 → C1 (stable) (78.1%)** C1 → C2 (7.8%) C1 → C3 (6.9%) C1 → C4 (5.8%) C1 → C5 (1.4%) C2 → C1 (5.1%) **C2 → C2 (stable) (70.8%)** C2 → C3 (13.6%) C2 → C4 (5.2%) C2 → C5 (5.3%) C3 → C1 (7.7%) C3 → C2 (6.2%) **C3 → C3 (stable) (78.8%)** C3 → C4 (6.7%) C3 → C5 (0.6%) C4 → C1 (5.0%) C4 → C2 (14.5%) C4 → C3 (12.2%) **C4 → C4 (stable) (61.0%)** C4 → C5 (7.3%) C5 → C1 (0.0%) C5 → C2 (4.5%) C5 → C3 (3.3%) C5 → C4 (16.3%) **C5 → C5 (stable) (75.9%)**		
Leech et al. (2015) (Older children) ***	C1: “Mostly healthy (41%)”, *PA↑ EDC↓ ST↓ TV↓* C2: “ED consumers who watch TV (32%)”, *EDC↑ TV ↑* C3: “High sedentary behavior/low MVPA (26%)” *PA↓ ST↑*	C1: “Mostly healthy (29%)”, *PA↑ EDC↓ ST↓ TV↓* C2: “ED consumers who watch TV (29%)”, *EDC↑ TV ↑* C3: “High sedentary behavior/low MVPA (42%) *PA↓ ST↑*	**C1 → C1 (stable) (56%)** C1 → C2 (8%) C1 → C3 (10%) C2 → C1 (1%) **C2 → C2 (stable) (61%)** C2 → C3(11%) C3 → C1 (5%) C3 → C2 (1%) **C3 → C3 (stable) (78%)**		
Parker et al. (2022)	C1: “Sedentary gamers (17%)”, *PA↓ ST↑ VG↑* C2: “Inactives “(46%)”, *PA↓ ST↑ VG↓* C3: “Actives (37%)” *PA↑ ST↓ VG↓*	C1: “Sedentary gamers (15%)”, *PA↓ ST↑ VG↑* C2: “Inactives (48%)”, *PA↓ ST↑ VG↓* C3: “Actives (37%)” *PA↑ ST↓ VG↓*	**C1 → C1 (stable) (93.8%)** C1 → C2 (4.6%) C1 → C3 (1.6%) C2 → C1 (0.0%) **C2 → C2 (stable) (84.0%)** C2 → C3 (16.0%) C3 → C1 (0.0%) C3 → C2 (22.5%) **C3 → C3 (stable) (77.5%)**		
Sánchez‐Oliva et al (2020)	C1”Highly Sedentary (7.7%)”, *PA↓ SB↑* ^+^ C2: “Sedentary (34.4%)”, *PA↓ SB↑* C3: “Active (46.3%)”, *PA↑ SB↓* C4: “Highly Active (11.6%)” *PA↑* ^+^ *SB↓*	C1: “Highly Sedentary (8.3%)”, *PA↓ SB↑* ^+^ C2: “Sedentary (35.8%)”, *PA↓ SB↑* C3: “Active (44.3%)”, *PA↑ SB↓* C4: “Highly Active (11.5%)” *PA↑* ^+^ *SB↓*	**C1 → C1 (stable) (34.10%)** C1 → C2 (44.70%) C1 → C3 (16.50%) C1 → C4 (4.70%) C2 → C1 (11.40%) **C2 → C2 (stable) (63.70%)** C2 → C3 (22.50%) C2 → C4 (2.40%) C3 → C1 (3.90%) C3 → C2 (21.60%) **C3 → C3 (stable) (61.90%)** C3 → C4 (12.60%) C4 → C1 (0.00%) C4 → C2 (4.70%) C4 → C3 (57.00%) **C4 → C4 (stable) (38.30%)**	Positive Affect Negative Affect HRQoL	C3/C4 → C1/C2 associated with a significantly greater decrease in positive affect than C3/C4 → C3/C4 OR C1/C2 → C3/C4 C1/C2 → C3/C4 attenuated the decrease in HRQoL.

* When interpreting changes in prevalence note that Saldanha‐Gomes et al (2020) presents the total number of participants who changed clusters across time.

** D'Souza et al (2021) did not provide any analysis on health behavior cluster change.

*** When interpreting results from Leech et al (2015) note that all transition probabilities that are not highlighted in **bold** total to 100%.

MVPA Moderate‐vigorous physical activity, ↓ Low, SG Sugar product intake, ↑ High, FV Fruit and vegetable consumption, SP sleep, SG Sugary food consumption, ST Screen time, OP Outdoor Play, SB Sedentary behavior, SV Savory food consumption, ‐ Average, BMI Body mass index, TV Television viewing, OPA Outdoor physical activity, DT Diet, PA Physical activity, ↓^+^ Very low, 0 No significant association, VG Videogaming, ↑^+^ Very high, EDC Energy dense food/drink consumption, FS Fatty and salty food intake, NB nibbling, DA Dairy consumption, HRQoL Health‐related quality of life.

Seven studies (39%) examined trajectories of behavioral clustering over time^49^, ^51^, ^52^, ^53^, ^55^, ^57^, ^58^ (see Table 3). Five studies (71%) used group‐based trajectory analysis (GBTA),^49^, ^52^, ^53^, ^57^, ^58^ one study (14%) used parallel process growth mixture models (PPGMM),^51^ and one (14%) study used growth mixture modeling (GMM).^55^

**TABLE 3:** Health behaviour cluster trajectories, and associations with health outcomes. This table provides an in‐depth overview of studies in the ‘trajectories’ category.

Author (year)	Health behavior cluster trajectories (prevalence %)	Outcomes assessed	Results (outcomes)
**<5 years**			
Chia et al. (2024)	C1: “Consistently healthy (11%)”, *Unhealthy pattern ↓ (→) Healthy pattern ↑ (→)* C2: “Consistently unhealthy (18%)”, Unhealthy pattern *↑ (→)* Healthy pattern *↓ (→)* C3: “Mixed pattern (71%)”. *Unhealthy pattern ↓ (→)* Healthy pattern *↓ (→)*	BMI z‐score Abdominal circumference Sum of skinfolds Blood pressure (systolic) Blood pressure (Diastolic) Pre‐hypertension HDL cholesterol Fasting insulin Triglyceride HOMA‐IR Fasting glucose Fasting insulin Metabolic syndrome score Fatty liver index	C2 + % of pre‐hypertension, + levels of diastolic blood pressure, fasting insulin, HOMA‐IR, triglycerides and metabolic syndrome vs C3. C1 showed no differences in cardiometabolic outcomes compared to C3
Del Pozo‐Cruz et al. (2019) (Younger children)	C1: “Low activity‐low screen, (48.2%)”, *PA ↓ (→) ST ↓ (→)* C2: “Increasing activity‐low screen, (27.2%)”, *PA (↗) ST ↓ (→)* C3: “Low activity‐increasing screen, (24.6%)” *PA↓ (→) ST (↗)*	health‐related quality‐of‐life (HRQoL) Socio‐emotional outcomes (SDQ)	C2 + SDQ scores and +HRQoL C3 ‐ SDQ scores and ‐HRQoL
**5‐11 years**			
Del Pozo‐Cruz et al (2019) (Older children)	C1: “Low activity ‐ Low screen, (46.2%)”, *PA↓ (→) ST↓ (→)* C2: “Increasing activity ‐ Low screen, (29.1%)”, *PA (↗) ST ↓ (→)* C3: “Low activity‐ increasing screen, (24.7%)” *PA ↓ (→) ST (↗)*	health‐related quality‐of‐life (HRQoL) Socio‐emotional outcomes (SDQ)	C2 + SDQ scores and +HRQoL C3 ‐ SDQ scores and ‐HRQoL
Farooq et al (2021)	C1: “Inactive throughout (31%)”, *PA↓ (→) SB↑ (→)* C2: “Active during Childhood (54%)”, *PA↑ (↘) SB ↓ (↗)* C3: “Active throughout (15%)” *PA ↑ (→) SB ↓ (→)*	Fat mass index (FMI)	C1 + FMI at follow up compared to C3.
Gallant et al. (2020)	**Boys:** C1: “Complier (12.1%)”, *PA ↑ (→), ST ↓ (→)* C2: “Decliner (22.6%)”, *PA (↘), ST (↘)* C3: “Non‐complier (42.3%)”, *PA ↓ (→), ST↑ (→)* C4: “MVPA‐complier group (23.0%)” *PA ↑ (→), ST (↘)*		
	**Girls:** C1: “Complier (9.0%)”, *PA ↑ (→), ST ↓ (→)* C2: “Decliner (18.5%)”, *PA (↘), ST (↘)* C3: “Non‐complier (42.5%)”, *PA↓(→), ST↑ (→)* C4: “Screen‐complier (30.0%)” *PA (↘), ST ↓ (→)*		
Parker et al. (2021)	C1: “Stable MVPA and decreasing SED (4%)”, *MVPA (→) SB (↘)* C2: “Stable MVPA and increasing SED (3%)”, *MVPA (→) SB (↗)* C3: “Consistently higher MVPA (18%)”, *MVPA ↑ (→)* C4: “Stable low MVPA and slight increase in SED (75%)” *MVPA ↓ (→) SB (↗)*		
Wilhite et al, (2023)	**Boys:** C1: “Highly actives (33%)”, *LPA ↑ (→) MVPA (↗) SB ↓ (→)* C2: “Inactive‐sitters (54%)”, *LPA ↓ (→) MVPA (↘) SB ↑ (→)* C3: “Decreasing activity (13%)”, *MVPA ↑ (↘) LPA ↓ (→)*		
	**Girls:** C1: “Highly actives (24%)”, MVPA *↑ (→)* C2: “Inactive‐sitters (27%)”, *MVPA ↓ (→) SB ↑ (→)* C3: “Decreasing activity (5%)”, *MVPA ↑ (↘)* C4: “High sleepers (28%)”, SP *↑ (→)* C5: “Lightly actives (16%)”’ *LPA ↓ (↗) SB ↓ (→)*		
**12 – 18 years**			
Hanson et al. (2019)	**Males:** C1: “Decreasing activity, non‐walkers (23%)”, *PA (↘) WK↓ (→)* C2: Active, walkers (29%)”, *PA↑ (→) WK ↑ (→)* C3: “Decreasing activity, walkers (48%)” *PA (↘) WK↑ (→)*		
	**Females:** C1: “Decreasing activity non‐walkers, (32%)”, *PA (↘) WK↓ (→)* C2: “Active, walkers (17%)”, *PA↑ (→) WK ↑ (→)* C3: “Decreasing activity, walkers (51%)” *PA (↘) WK ↑ (→)*		

() Brackets denote changes of specific behaviors across time (For example: PA (↗) indicates increasing levels physical activity. While PA↑ (→) indicates high levels of physical activity which remain stable over time.

* Unhealthy pattern is characterized by high intakes of processed meat, fast food, sweet snacks, savory snacks, sugar‐sweetened beverages, and screen time.

** Healthy pattern is characterized by high intakes of fruit and vegetables, low screen time, and high moderate‐to‐vigorous physical activity, outdoor play, and participation in organized physical activity.

↓ Low, → Stable, ↑ High, ↗ Increasing, + Higher, ‐ Lower, +% Higher odds of, PA Physical activity, ST Screen time, SB Sedentary behavior, MVPA Moderate‐vigorous physical activity, LPA Light physical activity, ESB Educational sedentary behavior, SP sleep, SSB Social‐based sedentary behavior, PT Passive transport, ↘ Decreasing, WK Walking.

### 3.5 Characteristics of clusters

A ‘healthy’ cluster was defined by combinations of characteristically healthy behaviors (e.g. high physical activity, low sedentary behavior, good quality diet, sufficient sleep) while an ‘unhealthy’ cluster was defined by combinations of characteristically unhealthy behaviors (e.g. low physical activity, high sedentary behavior, poor quality diet, and insufficient sleep). Meanwhile, ‘mixed’ clusters were defined by one or more ‘healthy’ behavior coexisting with one or more ‘unhealthy’ behavior (e.g. high physical activity, and poor‐quality diet).

### 3.6 Transitions between clusters across time

Of the 11 studies (15 independent samples) which examined cluster transitions, 6 studies (55%) examined transitions between clusters of physical activity, sedentary behavior, and diet,^42^, ^46^, ^54^ four studies (36%) examined transitions between clusters of physical activity and sedentary behavior,^43^, ^48^, ^50^ and one study (9%) examined transitions between clusters of physical activity, sedentary behavior, and sleep^45^ (see Table 2).

In six independent samples (40%), baseline ages ranged from 5 to 11 years, in five independent samples (33%) baseline ages were under years, and in four independent samples (27%) baseline ages ranged from 12 to 18 years. In total, across all studies, 99 clusters were identified (47 at base line and 52 at follow‐up).

Of the eleven studies that examined cluster transitions, seven studies (ten independent samples) found that the number and types of cluster patterns observed in the data were the same across time, thus allowing tracking to be examined. Among these studies, 66 clusters were identified (baseline n = 32, follow‐up[s] n = 34). Of those, 25 were categorized as ‘healthy’ clusters (baseline n = 12, follow‐up[s] n = 13),^42^, ^43^, ^45^, ^46^, ^48^, ^50^, ^54^ 27 were categorized as ‘unhealthy’ clusters (baseline n = 13, follow‐up[s] n = 14),^42^, ^43^, ^46^, ^48^, ^50^, ^54^ and 14 were categorized as ‘mixed’ clusters (baseline n = 7, follow‐up n = 7)^45^, ^46^, ^48^ (see Table 2).

**FIGURE 3:**
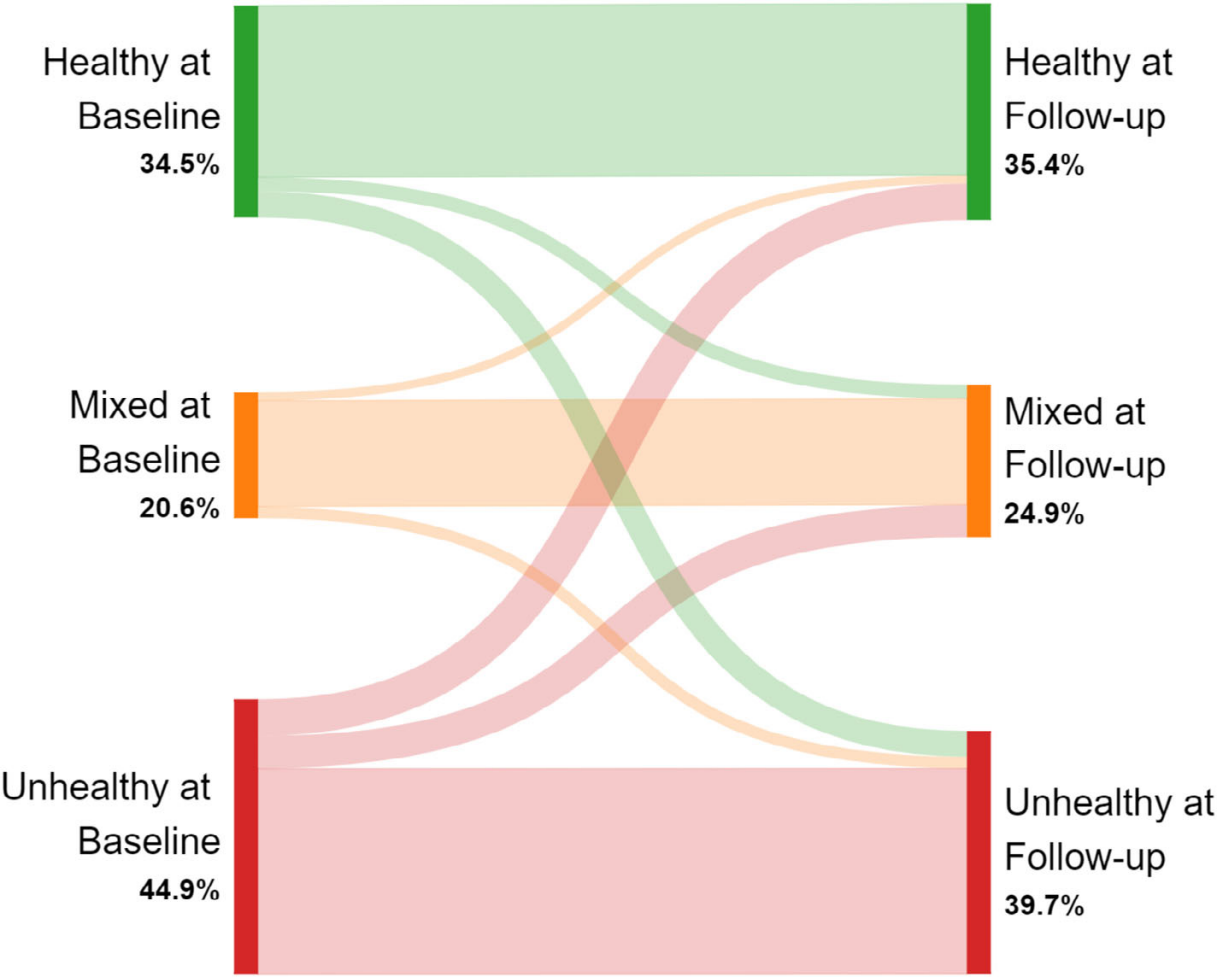
Sankey diagram titled ‘Transitions between clusters across time’. This diagram visualizes transitions between similar cluster types.

Six of these ‘tracking’ studies (86%) reported transitions through transition probabilities,^42^, ^43^, ^45^, ^46^, ^48^, ^50^ and one study (14%) reported tracking coefficients^54^ (see Table 2). In 91% (n = 31) of cases, the most common transitions involved participants remaining within the same cluster between baseline and follow‐up. Transition probabilities were reported for 28 clusters; 81% of participants who belonged to a ‘healthy’ cluster at baseline transitioned to another ‘healthy’ cluster at follow‐up, 84% of participants who belonged to a ‘mixed’ cluster at baseline transitioned to another ‘mixed cluster’ at follow‐up, and 75% of participants who belonged to an ‘unhealthy’ cluster at baseline transitioned to another ‘unhealthy’ cluster at follow‐up (see Figure 3).

Four studies (five independent samples) out of the 11 that examined transitions, found different types of clusters at different timepoints, a total of 33 clusters (15 at baseline and 18 at follow‐up) were identified. Among these, 13 ‘healthy’ clusters (baseline n = 6, follow‐up n = 7),^41^, ^44^, ^47^, ^56^ 12 ‘unhealthy’ clusters (baseline n = 6, follow‐up n = 6),^41^, ^44^, ^47^, ^56^ and 8 ‘mixed’ clusters (baseline n = 3, follow‐up n = 5)^41^, ^44^, ^47^, ^56^ were identified.

Three of these studies (four independent samples) examined transitions in 12 health behavior clusters through transition probabilities,^41^, ^44^, ^56^ while another did not report transition probabilities.^47^ Of the studies that reported transition probabilities, the most common changes observed were characterized by young people belonging to an ‘unhealthy’ cluster at baseline changing to another ‘unhealthy’ clusters at follow‐up (see Table 2).

### 3.7 Trajectories of clusters across time

Of the seven studies examining trajectories of behavioral clusters, one study (14%) examined clustering of physical activity, sedentary behavior, diet, and sleep,^58^ four studies (57%) examined clustering of physical activity, sedentary behavior, and sleep,^51^, ^57^ and one study (14%) examined clustering of physical activity and sedentary behavior^49^, ^52^, ^53^, ^55^ (see Table 4). A total of 39 trajectories were identified from the seven studies. In 57% (n = 4) of studies, baseline ages ranged from 5 to 11 years, in 29% (n = 2) participants were <5 years of age at baseline, and in 14% (n = 1) of samples baseline ages ranged from 12 to 18 years.

**TABLE 4:** Sociodemographic determinates of health behavior clustering across time. This table provides an in‐depth overview of studies in the ‘change’ category.

Socio‐demographic determinates	Related to cluster trajectories	Unrelated to cluster trajectories	No. of samples	Summary (n)	
	+	0		+	0
**Change**					
Gender	^44^ ^45^ ^46^		3	3	0
Ethnicity		^45^	1	0	1
Age	^46^		1	1	0
Post‐secondary education situational pathways.		^50^	1	0	1
Indicators of socio‐economic status	^46^	^44^	2	1	1
**Trajectories**					
Gender	^49^ ^55^	^58^	4	3	1
Ethnicity	^58^		1	1	0
Indigenous status	^55^		2	2	0
Indicators of socio‐economic status	^53^ ^55^ ^(^^Y)^ ^57^ ^58^	^55^ ^(O)^	7	6	1
Living with two biological parents	^55^		2	2	0
Mothers' country of birth	^53^		2	2	0
Childs birth order	^53^ ^(^^G)^	^53^ ^(^^B)^ ^58^	3	1	2
Number of siblings	^55^		2	2	0
English as first language	^55^		2	2	0

For references: See Table 1.

B Boys, G Girls, Y Younger sample, O Older sample.

**FIGURE 4:**
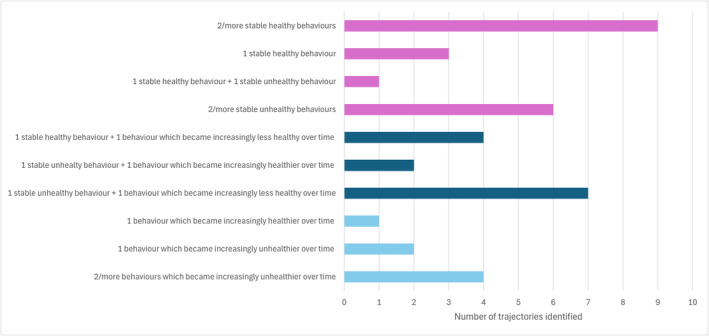
Bar chart titled ‘Defining characteristics of health behaviour cluster trajectories’. This diagram shows the prevalence of health behavior cluster trajectories with similar characteristics.

An overview of the trajectories of clusters over time is presented in Figure 4. The number of clusters observed in a single independent sample ranged from three^49^, ^52^, ^55^, ^57^, ^58^ to five.^57^ The most prevalent clusters identified nine times across all studies, were those defined by two or more co‐occurring healthy behaviors which remained stable over time.^49^, ^52^, ^53^, ^55^, ^57^, ^58^ Seven trajectories were defined by a single stable unhealthy behavior and a second behavior which became increasingly less healthy over time.^51^, ^52^, ^53^, ^55^, ^57^ Six trajectories were defined by two or more co‐occurring unhealthy behaviors which remained stable over time.^49^, ^52^, ^53^, ^55^, ^57^, ^58^ Four trajectories were defined by a single stable healthy behavior and a second behavior which became increasingly less healthy over time.^53^, ^55^ Four trajectories were defined by two or more behaviors which became less healthy over time.^49^, ^52^ Three trajectories were defined by a single healthy behavior which remained stable over time.^51^, ^57^ Two trajectories were defined by a single stable unhealthy behavior and a second behavior which became increasingly healthier over time.^52^, ^57^ Two trajectories were defined by a single behavior becoming less healthy over time.^51^, ^57^ One trajectory was defined by co‐occurring stable healthy and unhealthy behaviors across time.^58^ One trajectory was defined by a single behavior becoming healthier over time.^51^

### 3.8 Outcomes of clustering across time

Across all included articles, six studies examined associations between clustering across time and mental and physical health outcomes.^32^, ^41^, ^49^, ^55^, ^56^, ^58^ Three studies that examined transitions between clusters also examined associations between transitions and outcomes^41^, ^43^, ^56^ (see Table 2). Three studies that examined cluster trajectories also examined associations between cluster trajectories and outcomes^49^, ^55^, ^58^ (see Table 3).

### 3.9 Sociodemographic determinants of clustering across time

Across all included articles, nine studies examined sociodemographic determinants of behavioral clustering across time.^44^, ^45^, ^46^, ^49^, ^50^, ^53^, ^55^, ^57^, ^58^ Eleven socio‐demographic determinates were identified, with the majority (n = 8) of determinants assessed in single studies only (see Table 4). Six studies assessed differences in clusters according to socioeconomic status, with two studies identifying associations between lower socioeconomic status and an increased likelihood of belonging to a cluster trajectory defined by at least one unhealthy behavior which remained stable across time,^57^, ^58^ one study identifying an association between lower socioeconomic status and an increased likelihood of transitioning to a ‘mixed’ or ‘unhealthy’ cluster at follow‐up.^46^ Two studies identified associations between higher socioeconomic status and an increased likelihood of belonging to a cluster trajectory defined by unhealthy behaviors which remained consistent across time.^53^, ^55^ One study found no associations between socioeconomic status and clustering across time.^44^, ^55^

Six studies assessed differences in clusters according to gender. In two studies, girls were more likely than boys to experience unhealthy behavioral transitions,^44^, ^55^ and in different two studies, girls were more likely to remain within unhealthy clusters across time.^45^, ^49^ One study found that boys were more likely to exhibit consistently healthy behavioral clustering compared to girls,^45^ and one found that boys were more likely to exhibit unhealthy or mixed transitions compared to girls.^46^ One study found no association between gender and cluster transitions.^58^

Two studies examined differences in clustering according to ethnicity, with one finding no association^45^ and one finding that children of Malay ethnicity were more likely to belong to ‘consistently unhealthy’ cluster trajectories than children of Indian or Chinese ethnicity.^58^

## 4 DISCUSSION

This is the first systematic review to synthesize evidence from prospective studies examining the transitions and trajectories of clusters of physical activity, sedentary behavior, diet, and sleep through childhood and adolescence. Health behavior clusters identified in this review were classified as ‘healthy’, ‘unhealthy’, or ‘mixed’. Much of the evidence comes from studies of children aged 5‐11 years and suggests that health behavior clusters tended to track strongly across time. Among the studies where ‘tracking’ was examined, the most common transition observed (seen in 91% of cases) involved children remaining within the same cluster at baseline and follow‐up. Furthermore, among the studies which identified different types of clusters at different timepoints, half of all observed transitions occurred between clusters which shared similar characteristics such as moving from an ‘unhealthy cluster’ at baseline to another ‘unhealthy cluster’ at follow‐up. These findings highlight that children are establishing unhealthy behavioral habits at a young age, and that those unhealthy behaviors persist into later childhood and adolescence. Efforts are needed early in childhood to prevent unhealthy behaviors becoming habitual, to uncouple co‐exiting unhealthy behaviors, and to promote healthier lifestyle behaviors.

### 4.1 Overview of studies

Across 18 studies, the majority (89%) were published within the last five years, highlighting how recent the focus on behavioral clustering in prospective studies has been. Around half of the studies included in this review included data from children aged 5‐11 years at baseline, and most studies had a follow‐up period ranging from 2 to 3 years. Thus, younger children and older adolescents are not well represented in this review. All of the studies included in this review examined physical activity and sedentary behavior, while just under half included either diet or sleep variables. The comparative overrepresentation of studies exclusively examining physical activity and sedentary behavior has also been observed by other reviews which have primarily examined clustering cross‐sectionally.^28^, ^35^, ^59^ Diet is often overlooked with an increased focus on movement behaviors, encompassing physical activity, sedentary behavior, and sleep, following the development of 24‐hr movement guidelines in countries such as Canada and Australia.^60^, ^61^ Concerningly, an unhealthy diet is known to commonly cluster with high screen time in children and adolescents and may have adverse impacts on health outcomes, such as obesity.^62^, ^63^, ^64^, ^65^ By focusing on physical activity and sedentary behavior, researchers may inadvertently be overlooking the intricate interplay and synergistic effects added by diet and sleep, risking creating an oversimplified understanding of the clustering of health behaviors. Future interventions may be more effective at combating obesity and ill‐health, by targeting a wider combination of behaviors.

### 4.2 Clusters exhibit high levels of stability

The present review found that clusters of combinations of physical activity, sedentary behavior, diet, and sleep exhibit high levels of stability. Among the studies where ‘tracking’ was examined, further examination of transition probabilities reveals that a significant proportion of clusters exhibited a stable tracking probability ranging from 60% to 100%, suggesting that once children adopt certain health behavior patterns, these are highly likely to persist over time. These high tracking probabilities were present in all behavioral combinations assessed, and ‘unhealthy’, ‘mixed’, and ‘healthy’ clusters all showed similarly high levels of stability. Likewise, among studies examining transitions, where different types of clusters were found at different time‐points (i.e. studies which examined only change), the most common changes detected were those characterized by transitions between clusters which shared similar characteristics (i.e., moving from an ‘unhealthy cluster’ at baseline to another ‘unhealthy cluster’ at follow‐up. Findings from studies within the ‘trajectories’ category also indicated high consistency in behavioral patterning, with 41% of trajectory clusters being defined by two or more co‐occurring behaviors remaining stable across time. No discernible differences were identified in stability patterns by either baseline age or the make‐up of clusters (i.e. whether a cluster was created using all four behaviors or just physical activity and sedentary behavior variables). Together these findings suggest that both ‘healthy’ and ‘unhealthy’ health behaviors are deeply entrenched from childhood and highlight the challenges of instigating meaningful, sustained lifestyle changes. On the one hand, the findings are concerning as they indicate just how ingrained and pervasive unhealthy behaviors can be once they have become habitual. On the other hand, the findings offer promise in that healthy clusters also remain highly stable suggesting the possibility that if healthy behaviors can be established early, they may have a greater chance of persisting long‐term. The resilience of clusters across time underscores the importance of public health interventions focused on instilling healthy behaviors early in life which can provide a strong foundation for behavioral stability throughout life.

Future research should aim to explore the underlying mechanisms driving the high levels of cluster stability observed in this review. Consideration should be put towards understanding how, why, and in whom specific behaviors tend to cluster and which behaviors are most likely driving the persistence in the clusters over time. Understanding why and in whom health behavior clusters exhibit such resilience may help in the design of behavior change interventions. This review identified that few studies had examined socio‐demographic determinants of health behavior clusters across time, and that gender and indicators of socio‐economic position were the most commonly examined determinants. The evidence to date suggests that girls are more likely than boys to experience behavioral transitions characterized by the development of less healthy behaviors, and girls were more likely to remain within unhealthy clusters across time compared to boys. These findings are consistent with cross‐sectional evidence which suggests that girls are more likely to belong to ‘unhealthy’ or ‘mixed’ clusters, typically defined by low physical activity.^35^ However, it is important to note that while low activity characterizes these clusters for girls, other behaviors that typically accompany low activity (i.e. high sitting, poorer diet, and lower quality sleep) will be important to understand among girls. Respective explanations for these findings may be found in the observed trend that girls on average see a sharper decline in physical activity than boys as they get older^66^ and a noted gender‐based disparity in physical activity levels among young people, wherein girls are significantly less physically active than boys.^67^, ^68^ These findings suggest that girls are particularly at risk of getting trapped in unhealthy behavioral patterns or falling into less healthy profiles defined by low activity with age. Future research should investigate what drives these differences in gender seen in the changes of clustering.

Meanwhile, results regarding the association between indicators of socio‐economic position were mixed and suggest that the association is complex and may vary depending on the indicator of socioeconomic position assessed and the behaviors included in the cluster creation process. Our mixed findings also contrast with findings from previous reviews (which have primarily examined clustering cross‐sectionally) which suggest that young people from lower socio‐economic backgrounds exhibit unhealthier lifestyle patterns compared to those from higher socio‐economic backgrounds.^35^, ^59^ Further research that examines the same indictor and outcome combination is needed to build a strong evidence base and to identify potential target groups for intervention.

### 4.3 Clustering and health outcomes

Few of the included studies examined the associations between cluster transitions or trajectories and either mental or physical health outcomes. The most common outcomes were indicators of adiposity and weight status. Remaining within unhealthy clusters at multiple timepoints was consistently linked with higher BMI scores, higher body fat percentage, and greater waist circumference, signifying the risks of long‐term exposure to co‐existing unhealthy behaviors. Cross‐sectionally the evidence on the association between unhealthy behavioral clusters and markers of adiposity has been mixed^28^, ^59^ conflicting with the results of the current review. This discrepancy may be attributed to the difference in study design. These observations between cluster transitions and/or trajectories and obesogenic outcomes highlight the critical role of lifestyle behaviors in shaping long‐term health and the need for wholescale societal changes as the behaviors of interest here are often a result of the increasingly obesogenic environment.^69^, ^70^, ^71^ While indicators of adiposity serve as critical indicators of physical health, moving forward, further studies are warranted to broaden our understanding of a wider spectrum of potential health implications arising from long‐term adhesion to unhealthy behavioral trends. More studies examining the same domains of behaviors in association with further physical and mental health outcomes are needed.

### 4.4 Strengths and limitations

This is the first systematic review to synthesize the evidence on the evolution of clusters of physical activity, sedentary behavior, diet, and sleep through childhood and adolescence, their associations with mental and physical health outcomes, and the determinants associated with the transitions in clusters of behaviors. A key strength of this review is that it classified a heterogeneous assortment of studies examining health behavior clustering across time into discrete categories, allowing for clearer synthesis, and a more nuanced interpretation of findings.

Limitations, most of which are due to the literature itself, of this review must be considered when interpreting the results. There was considerable diversity in the measures and methods used to create clusters across studies, but the consistency in the trends observed instils a degree of confidence in the strength of the trends we have observed. Most studies had a high risk of bias, and most studies relied on self‐report tools for assessing the behaviors of interest. Self‐report tools though often reliable and effective at providing useful contextual information, do not to fare well in risk‐of‐bias assessments, such as the one used by this review. No study identified in this review had a follow‐up period longer than 3 years, therefore we cannot draw conclusions about longer‐term cluster stability. Further prospective cohort studies with longer follow‐up periods are required to address these questions. The heterogeneity in the measures and methods of assessing behaviors, and the ways in which clustering was analyzed, precluded a meta‐analysis from being conducted.

When interpreting results, it is also important to acknowledge the limitations of using data‐driven approaches to determine behavioral clusters. Most of the studies involved in this review provided details on the procedures and statistical indicators used to determine the optimal number of cluster solutions. However, this process still involves some level of subjectivity. It is crucial for researchers to transparently report how they interpreted any statistical indicators employed to justify their cluster solutions. Additionally, the clusters identified in these studies are not generalizable to other populations, as the findings are highly dependent on the specific dataset and the discrete behavioral variables inputted into the models. The adoption of a standardized, evidence‐based approach to clustering ‐ e.g. using a priori “lifestyle” scores ‐ could enhance the comparability of research findings across different populations and allow for more meaningful comparisons to be drawn.

## 5 CONCLUSIONS

This review found that ‘healthy’, ‘mixed’, and ‘unhealthy’ behavioral clusters all demonstrated high levels of stability suggesting that both positive and negative behavior patterns can become deeply entrenched and resistant to change. These findings emphasize the formidable challenge of instigating and maintaining long‐term behavior change. Concerningly, our review also found that remaining within unhealthy clusters at multiple timepoints was consistently linked with negative obesity‐related outcomes, underscoring the risks of long‐term persistence with multiple co‐occurring unhealthy behaviors. Overall, these findings highlight the critical importance of early interventions aimed at disrupting ingrained health behavior patterns. By understanding which factors influence the persistence of these clusters, efforts can be better directed toward developing effective, targeted strategies for behavior change that promote long‐term health and wellbeing. Moving forward, further attention should be directed towards exploring the nuances of behavioral patterning across diverse population subgroups.

## AUTHOR CONTRIBUTIONS

FB, NP & EH conceived the study. FB, EH, and NP developed the search strategy. FB conducted searches. FB, APS & NP screened all electronic articles. FP & NP conducted the full‐text review, and EH resolved conflicts. FB & APS carried out the risk of bias assessment, and NP resolved conflicts. FB extracted data. FB drafted the manuscript and EH, APS, & NP reviewed and edited the manuscript. All authors read and approved the final manuscript.

## CONFLICT OF INTEREST STATEMENT

The authors declare that they have no competing interests.

## CONSENT FOR PUBLICATION

Not applicable.

## AVAILABILITY OF DATA AND MATERIALS

The search strategy can be found in the supplementary information files.

## Supporting information


**Supplementary file 1:** Pubmed specific search strategy.
