# Supplementary material for: Tracking and changes in the clustering of physical activity, sedentary behavior, diet, and sleep across childhood and adolescence: A systematic review
Source: Obes Rev. 2025 Feb 18;26(7):e13909. doi: 10.1111/obr.13909 (PMC12137043; doi:10.1111/obr.13909)
Supplement: Supplementary file 1 — Pubmed specific search strategy. [file OBR-26-e13909-s001.docx]

Tracking and changes in the clustering of physical activity, sedentary behaviour, diet and sleep across childhood and adolescence: A systematic review

Finn Blyth1 , Emma Haycraft1 , Africa Peral-Suarez1, 2, Natalie Pearson1 1 School of Sport, Exercise & Health Sciences, Loughborough University; 2 Department of Nutrition and Food Sciences. Universidad Complutense de Madrid, Madrid, Spain.

Corresponding author: Finn Blyth. School of Sport, Exercise & Health Sciences, Loughborough University, Epinal Way, Loughborough LE11 3TU, Leicestershire, UK: [f.blyth@lboro.ac.uk](mailto:f.blyth@lboro.ac.uk)

**Supplementary file 1:**

**Pubmed specific search strategy**

(Infant [Mesh Terms] OR pre-school*[Title/Abstract] OR kindergar*[Title/Abstract] OR “young child*”[Title/Abstract] OR Child [MeSH Terms] OR child*[Title/Abstract] OR youth[Title/Abstract] OR “young people”[Title/Abstract] OR “young person*”[Title/Abstract] OR schoolchildren[Title/Abstract] OR school-age*[Title/Abstract] OR school-children[Title/Abstract] OR "school children"[Title/Abstract] OR boy[Title/Abstract] OR boys[Title/Abstract] OR girl*[Title/Abstract] OR (Adolescent [MeSH Terms] OR adolesc*[Title/Abstract] OR teen*[Title/Abstract]) AND (Health Behavior[MeSH Terms] OR "health behavio*"[Title/Abstract] OR lifestyle[Title/Abstract] OR life-style[Title/Abstract] OR "life style"[Title/Abstract] OR Physical activity [MeSH Terms] OR "physical activity"[Title/Abstract] OR "movement behavio*"[Title/Abstract] OR exercis*[Title/Abstract] OR sport*[Title/Abstract] OR sedentary lifestyle[MeSH Terms] OR "sedentary behavior*"[Title/Abstract] OR "sedentary behaviour*"[Title/Abstract] OR "sedentary lifestyle"[Title/Abstract] OR sedentary[Title/Abstract] OR "screen time"[Title/Abstract] OR screentime[Title/Abstract] OR screen-time[Title/Abstract] OR “screen media” [Title/Abstract] OR “screen viewing” [Title/Abstract] OR screen*[Title/Abstract] OR sitting[Title/Abstract] OR inactive*[Title/Abstract] OR Diet, Food, and Nutrition[MeSH Terms] OR diet*[Title/Abstract] OR “dietary behavio*”[Title/Abstract] OR “dietary intake*”[Title/Abstract] OR Eating[Title/Abstract] OR “eating behavio*”[Title/Abstract] OR “eating habit*”[Title/Abstract] OR “food intake”[Title/Abstract] OR nutrition*[Title/Abstract] OR “food intake”[Title/Abstract] OR “food frequency”[Title/Abstract] OR “energy-dense”[Title/Abstract]OR “healthy food”[Title/Abstract] OR fruit[Title/Abstract] OR “sugar-sweetened beverage*”[Title/Abstract] OR “snack*”[Title/Abstract] OR “sugar sweetened beverages”[Title/Abstract] OR “energy dense”[Title/Abstract] OR “nutritious food” [Title/Abstract] OR “nutrient-dense”[Title/Abstract] OR “nutrient dense”[Title/Abstract] OR Sleep [MeSH Terms] OR sleep*[Title/Abstract]) AND (Cohort studies [Mesh Terms] OR track*[Title/Abstract] OR longitudinal[Title/Abstract] OR Trajector*[Title/Abstract] OR pattern*[Title/Abstract] OR cohort[Title/Abstract] OR prospective[Title/Abstract] OR trend*[Title/Abstract]) AND (Cluster Analysis[MeSH Terms] OR Cluster*[Title/Abstract] OR Combination*[Title/Abstract] OR co-occur*[Title/Abstract] OR latent[Title/Abstract])
